# Delivering community-led integrated HIV and sexual and reproductive health services for sex workers: A mixed methods evaluation of the DIFFER study in Mysore, South India

**DOI:** 10.1371/journal.pone.0218654

**Published:** 2019-06-21

**Authors:** Sushena Reza-Paul, Lisa Lazarus, Raviprakash Maiya, K. T. Venukumar, Bhagya Lakshmi, Anuradha Roy, Partha Haldar, Michele Andina, Yves Lafort, Robert Lorway

**Affiliations:** 1 Centre for Global Public Health, Department of Community Health Sciences, University of Manitoba, Winnipeg, Canada; 2 Ashodaya Samithi, Mysore, India; 3 Centre for Community Medicine, All India Institute of Medical Sciences, New Delhi, India; 4 Department Uro-Gynaecology, International Centre for Reproductive Health, WHO Collaborating Centre, Ghent University, Ghent, Belgium; Simon Fraser University, CANADA

## Abstract

**Introduction:**

Women in developing countries continue to face barriers to accessing sexual and reproductive health (SRH) services, with marginalized women facing increased challenges to accessing care. The Diagonal Interventions to Fast-Forward Enhanced Reproductive Health (DIFFER) project implemented a package of interventions for female sex workers and women from the general population which integrated horizontal health services for the general population with existing vertical targeted interventions aimed at sex workers with an aim to improve SRH and HIV services. We present an outcome evaluation of the DIFFER project in terms of uptake rates for SRH services among sex workers in Mysore, India.

**Methods:**

Ashodaya Samithi, a sex worker-led organization, implemented the DIFFER strategy through their community-based clinic and a Well Women Clinic (WWC), established at a partner private hospital that provided SRH services for women living with HIV. Mixed methods were used to evaluate the intervention that included a baseline (2012–13) and end of project (2015–16) cross sectional surveys (CSS), focus group discussions (FGDs), key informant interviews, and analysis of service statistics from 2013–2016.

**Results:**

The CSS found that condom use, STI testing, and treatment were high before, and throughout the intervention; cervical cancer screening and treatment increased significantly, from 11.5% to 56% (aOR 9.85, p<0.001) and HIV testing in the last 3 months increased from 26.3% to 73.3% (aOR 7.25, p<0.001). The proportion of sex workers using any SRH service in the past year doubled from 25.7% to 51.4% (aOR 2.91, p<0.001). Service statistics showed similar trends. The FGDs and key informant interviews showed that women and stakeholders held high levels of satisfaction with the strategy, and affirmed potential for scale up.

**Conclusion:**

The DIFFER strategy demonstrated that SRH service uptake can occur in conjuction with HIV services offered to sex workers. This model of integrated service delivery has been accepted by policy makers and needs further analysis for scaling up.

## Introduction

Interventions for female sex workers globally have largely focused on ‘vertical’ approaches that directly target sex workers to address HIV and STI prevention, testing, and treatment [1–5]. Sociopolitical structural barriers, including the criminalization of sex work and HIV non-disclosure and occupational stigma and discrimination by healthcare providers [6–8], have prevented sex workers from being able to access health services aimed at the general population [9]. These targeted interventions designed for female sex workers mostly offer condoms and STI services and have rarely offered sexual and reproductive health (SRH) services, such as various forms of family planning services, care for unwanted pregnancies, and cervical cancer screenings [10,11]. As a consequence, sex workers have a broad range of unmet sexual and reproductive health needs [12–15] and existing studies have shown that sex workers shoulder the burden of large SRH disparities [6]. For example, a systematic review of facility-based sexual and reproductive health services for female sex workers in Africa found that cervical cancer screening was rare, despite a higher prevalence of abnormal cervical cytology than the general population [11].

In many countries, female sex workers continue to experience high rates of reproductive and sexual health morbidity and poor access to SRH services [16]. In India, for example, there is currently no national screening program for cervical cancer [17], despite calls for achieving “universal access to reproductive health” by 2015 in the Millennium Development Goals (MDGs) [18–19]. The MDGs have been criticized for having perpetuated a siloed approach to health care [20] and “may have exacerbated the divide between HIV and sexual and reproductive health (SRH) both by the adoption of a narrow vision of SRH only as maternal health and of HIV as only an infectious disease, ignoring its roots in human sexual behavior, and by separating HIV and SRH under different MDGs with distinct targets and indicators aimed at measuring solely these dimensions” [18]. The Sustainable Development Goals (SDGs), which were adopted in 2015, have worked to rectify this by including child and maternal health, as well as HIV and STI care under the same goal of “Good Health and Well-Being” (SDG 3)[21].

Access to SRH services remains a central aspect of women’s reproductive rights. However, the health disparities and barriers to care experienced by sex workers highlights the need for appropriate and nonjudgmental care that can promote better access to SRH services [6]. Successful strategies to improve sex workers’ access to HIV prevention, testing, and treatment have been based on community mobilization and empowerment models led by sex workers and tailored to meet their needs [22–25]. Similar strategies may help scale up access to SRH services. The Diagonal Interventions to Fast-Forward Enhanced Reproductive Health (DIFFER) project implemented a package of interventions for female sex workers and women from the general population which integrated horizontal health services for the general population with existing vertical targeted interventions aimed at sex workers with an aim to improve SRH and HIV services. We present an outcome evaluation of the DIFFER project in terms of uptake rates for SRH services among sex workers in Mysore, India.

## Methods

### Study site

In India, research was conducted in Mysore, a district in southern Karnataka. Mysore City and its surrounding areas has a population of over 920,00 according to 2011 Census data [26]. Health facilities include one district medical college hospital, three government-run HIV Integrated Care and Treatment Centres, an ART centre, and a maternity hospital. According to the National AIDS Control Program, Mysore is a high priority district (category A), meaning that it has had a higher than 1% HIV prevalence among antenatal care (ANC) populations in any of the sentinel surveillance sites for the last three years [27]. The city of Mysore has close to 3000 sex workers, of which 2000 are women and the rest are male and transgender sex workers. Three rounds of Integrated Behavioral and Biological Assessment (IBBA) conducted among women sex workers (2004–2009) documented HIV prevalence reducing from 25% to 11% [23,24]. The baseline cross sectional survey for DIFFER reported a total of 56 (12%) sex workers to be positive of the total 458 survey participants [28].

The project in Mysore was implemented by Ashodaya Samithi, a sex worker-led organization formed out of the social justice aspirations of women, men, and transgender sex workers. Since 2004, Ashodaya has been implementing HIV prevention programs with support from *Avahan*, the HIV/AIDS initiative of the Bill and Melinda Gates Foundation. Since 2012, the program transitioned to the Government and since then, Ashodaya has been implementing government supported HIV prevention programs in four districts in the State of Karnataka with a membership of 8000 sex workers. Ashodaya’s core areas of work include HIV/STI program implementation, including clinical services and outreach, advocacy, addressing sexual and gender-based violence, financial empowerment, capacity building and participatory research [23, 29–36]. Over the years, Ashodaya has built strong relations with important stakeholders, including local police, government, non-government agencies, academic institutions, legal authorities and policymakers at the district, state, and national levels. A team of community members has been trained as community researchers. Ashodaya has been designated as both a national and global learning site, resulting from their work on building the capacity of other sex worker organizations. Community engagement forms a crucial backdrop for interpreting the findings generated through research implemented by this site. However, despite these efforts, access and utilization of reproductive health services remain a challenge, as the government system has been unable to cater to the needs of sex workers.

### The DIFFER study

DIFFER was a multi-site study conducted in Durban, South Africa; the Tete-Moatize, Mozambique; Mombasa, Kenya; and Mysore, India [9,28,37–38]. In this study, we designed an intervention based on a needs assessment and a baseline Integrated Biological and Behavioural Assessment (IBBA) that pertain to service availability and acceptability. The strategy adopted was a ‘*diagonal’* one, incorporating a ‘*horizontal*’ health systems strengthening with more ‘*vertical*’ approaches. Horizontal reproductive health services are those that are normally available to the general population and provided as standard care through various government facilities. Vertical programs target specific populations who may be difficult to reach through a horizontal approach, such as sex workers. The DIFFER project was based on the hypothesis that combining vertical and horizontal services would be more effective, accessible, and cost-effective than providing them separately.

#### DIFFER intervention package in Ashodaya

Following a situation assessment, the intervention package was developed. This included: i) strengthening existing community mobilization & peer outreach; ii) strengthening existing HIV/STI services offered at the Ashodaya clinic and introducing visual inspection using acetic acid (VIA) screening and referral for cervical cancer; iii) introducing long-acting family planning methods (such as injectable birth control Depo-Provera) and increasing counseling focused on reinforcing the use of condoms for dual protection at the Ashodaya clinic; iv) referrals and linkages with government hospitals by health care navigators, v) preventing sexual and gender-based violence; and vi) initiating a “Well Women Clinic” at Asha Kirana (a hospital for people living with HIV) to integrate SRH services for HIV positive women. Importantly, the intervention package drew on funding from the existing targeted intervention and did not require the addition of staff. Instead, existing Ashodaya clinical staff received training to provide the new services related to VIA screening and other family planning methods, while the established network of health care navigators ensured referral and follow up at partner tertiary care hospitals. All services, except emergency contraception, were provided free of cost to participants. Emergency contraception pills, which are also not provided free of cost from government centres, were provided by Ashodaya at a discounted price.

We implemented the DIFFER strategy through a multi-pronged approach. Mixed methods were used to evaluate the intervention that included a baseline (2012–13) and end of project (EOP) (2015–16) cross-sectional surveys (CSS), focus group discussions (FGDs) at the same time points, and key informant interviews. These data were triangulated with the service statistics obtained from the Ashodaya clinic and Well Women Clinic (WWC) for the intervention duration (2013–2016).

The quantitative outcome indicators that were examined in this study were use of HIV/SRH commodities and services by sex workers that include condom use, STI care, HIV testing and care, use of contraception, and cervical cancer screening and care. Qualitative outcome data focused on the acceptability and awareness of the intervention, the feasibility of the integrated model, and its sustainability and scalability.

Ethics was obtained from Ghent University in Belgium and from the Asha Kirana Institutional Ethics Committee (IEC), Mysore, India. Informed written consent was obtained for all participants. The findings have also been previously described in the multi-sited final report [27].

### Data collection

#### Cross-sectional survey among sex workers

A representative sample of sex workers, sufficiently large to allow the measurement of changes in key variables between the pre-and post-intervention surveys, was recruited using Respondent-Driven-Sampling (RDS) [39–40]. RDS was chosen because sex work in Mysore is network-based and dispersed. Approximately 1800 female sex workers were using the services. The sample size was calculated to be 400 sex workers to allow for the detection of significant changes in key project indicators between the initial baseline survey and the end of project survey. To ensure inclusion of all sub-populations, a total of 8 seeds were selected from various sex work networks. Each was then given 5 coupons to distribute to potential participants. Based on previous studies, Ashodaya determined that 3 waves were optimal for the sex work context in Mysore. We recruited 458 participants at baseline and 415 sex workers participated in the EOP (2015–2016) survey.

The questionnaire was translated into the local language, Kannada, and back translated to English to ensure the integrity of the questions. Trained community researchers who are not part of the DIFFER team conducted the face-to-face interviewers using paper-based questionnaires. All participants were female, 18 years or older, received money or gifts for sex at least three times in the last six months, and had to be capable and willing to provide informed consent to participate. SPSS 21 was used to do the initial descriptive analysis. The coupon data were merged with the interview data and exported to Stata (Version 14.2, College Station, TX). We calculated prevalence estimates, adjusted for the unequal probability of inclusion due to varying social network sizes and the similarities in characteristics of people within social networks, using the Stata RDS analysis package and the ‘Volz-Heckathorn’ estimator. Changes in uptake of SRH services between the two surveys were assessed for statistical significance by merging the baseline and end EOP data sets, and fitting a logistic regression model with care seeking as a dichotomous variable (1 = sought care, 0 = did not seek care), RDS-adjusted weights, and using jack-knife resampling.

#### Focus group discussions

Focus groups discussions (FGDs) were conducted with sex workers at baseline and EOP. Participants were selected via purposive sampling. Six FGDs were conducted from January to March 2013 and 8 took place from January to March 2016. Each FGD included approximately 8–10 members. FGDs were facilitated in Kannada by community researchers and non-sex work Ashodaya program staff, audio recorded, and transcribed in English. FGD participants completed a brief socio-demographic survey. The FGD guide focused on: knowledge and use of SRH services; access to SRH services; stigma and discrimination faced by the community; outreach; community mobilization; and satisfaction with DIFFER services (for EOP FGDs). Transcripts from the focus groups were coded for key themes and emergent categories, followed by thematic and content analyses. Selected quotations were highlighted to illustrate the main themes. The qualitative outcome data was used to further contextualize the quantitative outcomes.

#### Key informant interviews with stakeholders

Key informant interviews were also conducted at baseline and EOP. Eligibility criteria included involvement with policy development, part of health service delivery at district, state, or national level, involvement with sex workers, and/or with DIFFER (especially at EOP). Participants included policymakers, government representatives from the district, state, and national levels with experience in departments ranging from the Ministry of Health and Family Welfare, the National AIDS Control Organization (NACO), Reproductive and Child Health, Department of Women and Child Development, leaders from NGOs working with sex workers in Mysore, lawyers who work as advocates for sex workers and sex worker leaders. Participants were purposively sampled to ensure representation of community leaders and different partners at local, state, and national levels. Interviews were conducted either in person, and were audio recorded and transcribed into English, or via phone, in which case extensive notes were taken during and immediately after the conversation. Key Informant interviews focused on the feasibility, appropriateness, and sustainability of the interventions. Most key informants were met individually, however a few group discussions took place. Consent was obtained prior to the discussions. Interviews were conducted by Ashodaya staff and researchers. Detailed notes were taken immediately following the interviews. Interview notes were then manually coded for emergent themes.

#### Service statistics from the clinic data

Clinic data was collected between October 2013 and September 2016. Clinic data collection began in 2013, as 2012 was the preparatory planning period for the multi-site intervention. The main service provider for sex workers was the Ashodaya clinic in Mysore city. For general population women, Ashodaya worked with Asha Kirana Hospital where the situation assessment documented the need for SRH services for women living with HIV and services were provided through the Well Women Clinic.

## Findings

### Quantitative study components

In this section we present our findings from two rounds of CSS and Ashodaya clinic service statistics (among sex workers) collected between October 2013 and September 2016. Subsequently, we present service statistics from the Well Women Clinic at the Asha Kirana hospital.

#### Socio-demographic and sex work characteristics

At baseline, 458 sex workers completed the CSS, with 415 participating at EOP. The age distribution was similar between the two surveys, with women reporting a median age of 34 (range 18–48) at baseline and 32 (range 18–45) at EOP. In both the surveys, most women (70.8% EOP vs. 77.2% baseline) reported being married. Sex work characteristics were similar between the two surveys (Table 1). The median number of commercial sex acts at EOP was 1 a day and 18 in the past month. At baseline, they were 2 and 20, respectively. The amount charged per sex act remained consistent at 500 INR. However, 33.4% reported to have other source of income at EOP compare to 27.8% at baseline.

**Table 1:** Socio-demographic and sex worker related characteristics of participants–between baseline and EOP CSS.

Characteristic	Unadjusted estimates				RDS adjusted estimates			
	Baseline		EOP		Baseline		EOP	
	n	%	n	%	%	95% CI	%	95% CI
**Age (years)**								
Median (IQR)	34		32					
Range	18–48		18–45					
< = 25	64	14	62	15	16.9	11.4–24.2	18.1	9.3–29.5
26–35	243	53	208	50.2	52.5	34.5–67.3	46.4	33.6–67.5
> = 36	151	33	145	34.9	30.7	23.2–39.2	35.6	27.5–43.5
**Education**								
Less than primary	367	80.1	202	48.7	79	67.4–87.7	54.8	45.7–63.3
Primary completed	51	11.1	192	46.3	16.7	8.1–27.8	18.8	11.7–27.4
Secondary completed	40	8.7	21	5.1	4.3	2.3–7.0	26.4	19.4–33.6
**Present relationship**								
Unmarried	32	7	27	6.5	8.9	3.6–16.6	13.6	6.2–21.5
Married	346	75.5	315	75.9	77.2	68.6–84.4	70.8	62.6–79.6
Widowed/Divorced	59	12.9	73	17.6	13.9	9.3–19.7	15.6	10.9–20.4
Refused	21	4.6	-	-	-	-	-	-

#### Use of HIV commodities and services by sex workers

Table 2 presents the unadjusted and adjusted results of the use of different HIV prevention and care commodities and services, as reported by the interviewed sex workers in the baseline and EOP surveys.

**Table 2:** Use of HIV services and related commodities by participants–RDS effect—Comparison between 1st and 2nd CSS.

Characteristic	Unadjusted estimates				RDS adjusted estimates						
	1st CSS		2nd CSS		1st CSS		2nd CSS		OR	95% CI	p-value
	N	n(%)	N	n(%)	N	n(%)	N	n(%)			
**Always used condoms in past month with any client**	458	431(94.1)	415	405(97.6)	458	441(96.2)	415	407(98.1)	1.67	0.47–5.92	0.427
**Always uses condoms with all partners** **(N: Excludes women who desire pregnancy)**	441	294(66.6)	410	319(77.8)	441	238(53.9)	410	217(53.0)	0.75	0.43–1.29	0.296
**Abnormal discharge or genital ulcer in past year**	458	143(31.2)	415	116(28.0)	458	159(34.8)	415	93(22.3)	0.58	0.28–1.02	0.058
**Care sought for last STI/RTI syndrome** **(N: Had discharge or ulcer in past year)**	143	119(83.2)	116	98(84.5)	143	106(74.4)	116	65(55.8)	0.44	0.10–1.95	0.281
**Ever tested for HIV**	458	442(96.5)	415	412(99.3)	458	436(95.2)	415	406(97.8)	2.31	0.20–26.6	0.502
**When last tested for HIV** **(N: Has not had a previous positive HIV test)**											
Less than 3 months	428	133(31.1)	381	287(75.3)	428	113(26.3)	381	279(73.3)	7.25	3.94–13.4	<0.001
Less than 6 months	431	226(52.4)	382	335(87.7)	431	175(40.5)	382	334(87.4)	9.9	5.27–18.6	<0.001
Less than 12 months	432	384(89.0)	385	379(98.4)	432	332(76.8)	385	368(95.7)	6.83	2.11–22.1	0.001
**Tested positive at last HIV test** **(N: Ever tested for HIV)**	437	33(7.6)	412	38(9.2)	437	35(8.0)	412	43(10.4)	1.3	0.57–2.96	0.529
**Currently using HIV care services** **(N: HIV positive)**	33	29(87.9)	38	36(94.7)	33	31(92.7)	38	36(94.7)	1.71	0.07–43.7	0.741
**On ART**	33	27(81.8)	38	30(79.0)	33	31(92.8)*	38	30(79.0)	0.83**	0.23–2.98	0.776

*RDS adjusted proportion could not be calculated and the weighed proportion is shown instead.

**RDS adjusted proportion and weighed proportion could not be calculated and the non-adjusted/non-weighed proportion is shown instead.

**Condom use**: Condom use has been a key focus of the Ashodaya intervention even before DIFFER was initiated and continued to be during this project as well [23]. Under the DIFFER intervention, self-reported consistent condom use with any clients in the last month remained high and even slightly increased at EOP but was not statistically significant (96.2% vs. 98.1%, aOR 1.67, p = 0.427). Consistent condom use with all partners remained stable after adjusting the sampling bias (53.9% at baseline vs. 53.0 at EOP, aOR 0.75, p = 0.296).

**STI care**: Less participants had genital symptoms in the past year at EOP than at baseline (34.8% vs. 22.3%, OR 0.58, p = 0.058). The number of participants who reported to have sought care for these genital symptoms was slightly higher (83.2% vs. 84.5%), but lower when adjusting for the sampling bias (74.4% vs. 55.8%). While this was not statistically significant (p = 0.281), any interpretation needs to be handled with caution as the difference could be due to bias in response on network site or other reporting bias to this question. When seeking care, most women reported coming to Ashodaya for treatment in both CSS. Participants reported a 91.8% very satisfied rate with STI service delivery at the end of the project.

Clinic data shows that >90% of all the sex workers registered with Ashodaya (N = 1605) were coming to the clinic quarterly for STI screening in the form of speculum examination to identify asymptomatic STIs and bi-annually for syphilis screening. The total number of STI cases diagnosed using enhanced syndromic diagnosis and treated from October 2013 to September 2016 were 272. All these were treated and follow-up visits showed that in all cases symptoms were resolved.

**HIV testing and care**: The number of participants reporting to have ever been tested for HIV increased from 95.2% at baseline to 97.8% at EOP (aOR2.31, p = 0.502), and the numbers recently tested increased significantly. Almost three quarters (73.3%) reported to have been tested in the past 3 months, while at baseline this was only one quarter (26.3%, aOR7.25, p<0.001). Over 87.6% chose Ashodaya for testing at EOP (compared to 79.1% at baseline, aOR 1.85, p = 0.231) and over 87.6% expressed that they were very satisfied with the availability of HIV testing services. The proportion of participants reporting to be HIV positive is similar across the two surveys (8.0% at baseline vs. 10.4% at EOP, aOR 1.3, p = 0.529). The proportion reporting to be in HIV care was already very high at baseline (92.7%) and remained high (94.7%, aOR 1.71, p = 0.741). A lower proportion however reported taking ART at EOP (79% vs. 92.8% at baseline, OR 0.83, p = 0.776). This was mainly because at the time of the project, the ART program supported by government was guided by the CD4 count (<300). Therefore, when people were diagnosed early, it was quite possible that the CD4 count was high and therefore, they were not on ART, though all were registered.

The clinic data shows that during the project period (October 2013—September 2015), a total of 16 participants were newly diagnosed with HIV and a cumulative of 92 were registered to the ART centre. Among them, 43 are on ART (as per the treatment guideline requirement CD ≤300).

#### Use of SRH commodities and services by sex workers

Table 3 presents the unadjusted and adjusted results of the use of different SRH commodities and services, as reported by the interviewed sex workers in the baseline and EOP surveys.

**Table 3:** Use of other SRH commodities and services by sex workers—Unadjusted and adjusted for RDS effect-comparison between 1st and 2nd CSS.

Characteristic	Unadjusted data				Adjusted data for RDS effect						
	1st CSS		2nd CSS		1st CSS		2nd CSS		OR	95% CI	p-value
	N	n(%)	N	n(%)	N	n(%)	N	n(%)			
**Currently using contraception** **(N: Not wanting to get pregnant, not pregnant, and able to conceive)**	381	375(98.4)	396	396(100)	381	365(95.8)	396	396(100)	-	-	-
**Main contraception method used** **(N: Currently using contraception)**											
Injectable contraceptive	374	1(0.3)	396	12(3)	373	0(0)	396	10(2.5)	105	40.4–275	<0.001
Oral contraceptives	374	5(1.3)	396	23(5.8)	373	2(0.6)	396	44(11)	12	2.32–61.6	0.003
IUD	374	4(1.1)	396	1(0.3)	373	4(1)	396	1(0.3)*	0.27	0.06–1.31	0.105
Implant	374	1(0.3)	396	0(0)	373	1(0.3)	396	0(0)	-	-	-
Condom	374	23(6.2)	396	39(9.9)	373	37(10)	396	58(14.7)	1.44	0.16–13.0	0.747
Female sterilization	374	339(90.6)	396	321(81.1)	373	329(88.2)	396	283(71.5)	0.39	0.08–1.91	0.244
**Currently using a non-barrier modern contraceptive method**	379	351(92.6)	396	357(90.2)	379	323(85.1)	396	338(85.3)	0.97	0.21–4.47	0.964
**Ever used emergency contraception** **(N = Excludes those who didn’t respond)**	455	15(3.3)	415	18(4.3)	455	11(2.4)	415	28(6.7)	2.65	0.45–15.7	0.283
**Unintended pregnancy in the last five years**	458	43(9.4)	397	31(7.7)	458	37(8)	397	29(7.2)	0.88	0.41–1.88	0.752
**Action taken for unwanted pregnancy** **(N = Had unwanted pregnancy in the past 5 years)**											
Had abortion	43	38(88.4)	32	29(90.6)	43	40(93.7)	32	31(96.0)*	1.64	0.13–20.5	0.697
Kept the pregnancy	43	5(11.6)	32	3(9.4)	43	3(6.3)	32	1(4.0)*	-	-	-
**Ever tested for cervical cancer**	458	50(10.9)	415	302(72.8)	458	53(11.5)	415	232(56)	9.85	5.29–18.3	<0.001
**Ever tested for cervical cancer** **(N = Age> = 30 years)**	337	35(10.4)	232	169(72.7)	337	46(13.6)	232	140(60.5)	10.8	5.41–21.7	<0.001
**Used all SRH services needed** **(N = Those who are eligible for the questions use of a non-barrier contraception method, ever have been screened for cervical cancer if older than 30 years, and having sought medical care for last forced sex)**	428	89(20.8)	408	220(53.9)	428	110(25.7)	408	210(51.4)	2.91	1.63–5.20	<0.001
**Used all HIV/SRH services needed**	458	30(6.6)	415	123(29.6)	458	26(5.6)	415	91(21.9)	4.46	1.97–10.1	<0.001

* RDS adjusted proportion could not be calculated and the weighed proportion is shown instead.

**Contraception**: Contraception use among participants who do not want to become pregnant or are not pregnant but are able to conceive was already very high at baseline (95.8%) and was 100% at EOP. In the methods used, we observe a significant increase of the use of hormonal contraceptives (0.6% vs. 11%, aOR 12, p = 0.003). Emergency contraception was used slightly more at EOP than at baseline (6.7% vs. 2.4%, aOR2.65, p = 0.283), but still rarely used. The difference was not statistically significant. The majority of the participants (82.7%) expressed that they were very satisfied with the availability of contraceptive services. The proportion that reported to have had unwanted pregnancies in the past 5 years was similar across surveys (8% at baseline vs. 7.2% at EOP, aOR 0.88, p = 0.752). The proportion who said that they sought an abortion for their unwanted/unintended pregnancy at a health facility was already high at baseline and remained so at EOP.

**Cervical cancer screening**: Ever having been screened for cervical cancer increased five-fold between surveys and more than half of the participants now have ever been screened (EOP 56% vs. 11.5% baseline, aOR 9.85, p<0.001).

Table 4 documents the distribution of cervical cancer screening at the clinic. During the project period, a total of 2302 VIA tests were conducted among 1562 sex workers. VIA is the standard screening method in low-income countries and used to diagnose pre-cancerous or cancerous lesions for early treatment [41]. Only 836 had undergone testing once, with the remainder tested more than once during the project period. Among 1562 sex workers, 103 (6.6%) had reactive tests and among them, 39 (37.9%) were treated with medication and followed-up and 64 (62.1%) underwent biopsy. Only 3 people tested positive for biopsy and were treated as per the government protocol.

**Table 4:** Program data on cervical cancer tests in Ashodaya clinic (October 2013-September 2016).

Characteristic	n	%
**Number of VIA tests conducted**	2302	
**Number of individuals who have undergone VIA testing**	1562	
**Number of individuals tested only once**	836	
**Number of individuals tested more than once**	726	
**Number of VIA reactive**	103	6.59
**Number of VIA reactive treated and symptoms resolved with medication**	39	37.86
**Number of VIA reactive undergone biopsy**	64	62.14
**Number of reactive in biopsy test**	3*	4.69

* All have undergone surgery to remove uterus after the biopsy test

### Qualitative study components

#### Focus group discussions and key informant interviews

Following the DIFFER intervention, eight focus group discussions occurred. Women who participated in the FGDs were more aware of and had better access to SRH options at EOP. The analysis focused on the awareness/access, acceptability, and sustainability of the intervention. Key informant interviews were done with nine stakeholders at EOP and the interviews explored the themes of acceptability and need, and sustainability and scalability.

#### Socio-demographic characteristics

The information sheet (socio-demographic questionnaire) of the FGD participants revealed that the median age of the participants was 32.2 years and 55% were illiterate. Most of the participants (70.6%) worked in mixed settings of street and home. Almost 70% of the participants did not have any other source of income. The median number of children was two. The mean number of clients in the last month was reported to be 15 and that of non-paying partners was two.

#### Acceptability and awareness

*Since we are into sex work the chances are more for uterus related problems and it is a serious issue*, *so we need to get it treated*. *Therefore*, *we find these services as very helpful*.

[Focus group participant]

New SRH services increased knowledge of cervical cancer and access to testing among the FGD participants. Sex workers were able to come to Ashodaya to treat symptoms and were able to access screening and effective treatment for their cervical cancer. These new services also increased women’s own commitment to regular screenings.

*I came to Ashodaya clinic with a stomachache*, *was not aware of its seriousness*, *after necessary tests they found a [uterine] tumor and referred me to Asha Kirana hospital for operating the same*. *After the operation now*, *I make sure I get myself checked every month coming to the clinic here and am healthy and happy now*.

[Focus group participant]

Women expressed satisfaction with the new SRH services offered by Ashodaya and stated that they benefitted from this expansion of services.

*Earlier Ashodaya used to work upon the prevention of HIV and other STIs but now it is also working for reproductive health*, *which we find as a great improvement in the services provided to us*.

[Focus group participant]

Importantly, focus group participants discussed how Ashodaya’s system of health care navigators (also known as community volunteers) and referrals helped facilitate access to services outside of the Ashodaya clinic. Furthermore, health care navigators worked to minimize experiences of stigma and discrimination.

***Interviewer***: *Can you share your experience at [hospital name] like what kind of discrimination*?

***Respondent 1***: *They do not treat us well as we are sex workers*. *They leave us waiting for hours no matter how bad our position is*. *They don’t even bother about our sufferings*.

***Interviewer***: *Do you introduce yourselves as a sex worker*?

***Respondent 2***: *No*. *But they recognize*.

***Interviewer***: *We have deputed two volunteers at [hospital name]*. *Are you finding any change because of this from the past two years*?

***Respondent 3***: *Yes these volunteers help us availing the best services there and the hospital staff also treat us well*.

[Focus group participant]

Findings from the key informant interviews also found that sex workers appreciated the integrated service and the demand for new services continues.

*Ashodaya was only providing STI treatment and condoms*, *but now I started getting advice on pregnancy*, *got tested for cervical cancer*, *even counseling referral in case I needed an abortion*. *I feel happy my organization is giving me more health related services then before*….

[Key informant interview, sex worker from Ashodaya]

#### Feasibility of integrated model

While global health policy increasingly reiterates the importance of integrated service delivery, virtually none have actually documented "how to implement" such an intervention. Most stakeholders felt that this approach of integration has served well for sex workers and marginalized communities, as well as for women from the general population.

*Ashodaya’s work related to DIFFER has been timely and relevant*. *They provided messages on FP [family planning]*, *cervical cancer*, *abortion not only to their FSW [female sex worker] community but also to HIV-positive women… They promoted VIA*, *took special care for those who tested positive for subsequent follow-up*. *They even trained our doctors on VIA screening*. *I’m glad that they have provided services beyond HIV to sex workers… Earlier there was no model*, *no program has shown how to do this*. *Ashodaya has shown how all the services can be provided*…

*[Key informant interview*, *stakeholder*/*government policy maker]*

Ashodaya has demonstrated how to successfully integrate SRH and HIV service delivery. Strategic advocacy, which involved presenting findings from the DIFFER intervention to key stakeholders who are able to influence decision-making processes, elicited positive responses from them to provide integrated HIV/SRH services to sex workers, including screening, counseling, and treatment. Health managers, health providers, and community workers found the program to be very effective.

*It’s a very important project*. *With Ashodaya’s intervention*, *we started routine screening for cervical cancer for HIV+ women*. *We are seeing a lot of women being reactive for VIA*. *Early detection is leading to early treatment*. *We are happy that we are providing the services to them and they are happy not only for cancer screening but also for FP [family planning] services*.

[Key informant interview, Physician]

*Basically we would be able to provide add-on services*. *And when these services are provided*, *especially to persons with HIV*, *they will be far better than what they were*. *Any additional services like given*, *they [people] are benefited … Irrespective of funding or no funding*, *we wish to continue doing that*. *We have found it very beneficial to lots of HIV-positive women who come to our hospital*….

[Key informant interview, physician]

#### Sustainability and scalability

Strategic advocacy with the State AIDS Control Society (SACS) led to the development of integrated monitoring tools and fostered project ownership at both the state and district levels.

*During our visits*, *we worked out the monitoring tools with Ashodaya*. *We used our existing tools*. *We discussed with them about new indicators*. *It was very much possible to add new indicators in the existing formats…*. *so you see there are things which are readily available and can be used for scaling up*…

[Key informant interview, representative from SACS]

District and state level stakeholders considered DIFFER’s community-led integrated service delivery approach highly reliable. Most stakeholders felt this model should be scaled up and that Ashodaya could play an important role in this scale up.

*They’ve already trained our physicians*. *Based on Ashodaya’s experience*, *Ashodaya Academy [Ashodaya’s Learning and Training Site] can do a training program of CBOs [community-based organizations] and other organizations*, *which are implementing HIV programs in the state*. *KSAPS [Karnataka Sate AIDS Prevention Society] should advocate with NACO [National AIDS Control Organization] to integrate SRH and HIV programs… Send us your proposal*, *we will take it up*….

[Key informant interview, stakeholder from the State Government]

Importantly, the project occurred at a critical moment when NACO and the National Health Mission (NHM) are revising their SRH strategies.

*This is a critical time for TI [targeted intervention] funding*. *So*, *it’s appropriate to provide all services that can be delivered at the grassroots level in a comprehensive way*. *We are mobilizing the community for HIV services*, *and we should offer them other SRH services that they require*. *Or else we may see a huge rise in cervical cancer among FSWs…*.*Through this approach we can provide services under one roof*…

[Key informant interview, representative from State Government]

## Discussion

The DIFFER intervention demonstrated that SRH service uptake can occur in conjuction with HIV services offered to sex workers. The project appeared effective in scaling up access to cervical cancer screenings and treatment, injectable contraceptives, as well as maintaining an already well-established condom distribution system and STI/HIV screenings and treatment. Importantly, sex workers and key stakeholders were extremely satisfied with the provision of these new SRH services and have expressed an interest in further scaling up services.

Ashodaya’s intervention philosophy involved a flexible approach that prioritized the needs of sex workers and focused on identifying service gaps to move towards comprehensive service delivery, rather than single service care. This involved a move from vertical services to an integrated package of services. Ashodaya collaborated with their partners, such as Asha Kirana, on the intervention process. Extensive discussions with Ashodaya community members and the Ashodaya Board was an integral step in identifying priority issues. In this way, DIFFER built on a well-established community mobilization HIV/STI prevention model. Strategic advocacy at the State and District levels was conducted to integrate required services with the targeted intervention, and establish linkages with government and private service providers, including placement of Health Care Navigators.

Existing research has pointed to community empowerment and peer-led models as demonstrating successful strategies for scaling up interventions that address HIV and STI prevention and access to SRH services [1,29, 40, 42]. Furthermore, universal access to contraception and improving SRH services will be critical to achieve the Sustainable Development Goals [21]. There has also been recognition of the need to strengthen linkages between SRH and HIV prevention services for sex workers, rather than focusing solely on STIs and HIV prevention. In the 2015 National Health Policy, the Government of India has promised free comprehensive primary health care services for all aspects of reproductive, maternal, child, and adolescent health [43].

Ashodaya has integrated SRH and related services into their intervention and has advocated with KSAPS to develop indicators in their reporting formats to capture data on SRH, along with HIV data. Both the National AIDS Control Organization and National Health Mission have expressed interest in and support for moving this integrated HIV/SRH model forward. The Government of India has already mentioned in their National AIDS Control Program IV strategy document that SRH will be integrated along with HIV prevention, treatment, care, and support [43]. Accordingly, there are processes in place at the national level to develop a comprehensive SRH package and protocols and build capacities of healthcare providers, including counselors, to roll out the integration.

It must be noted that the success of scaling up an integrated HIV/SRH intervention is dependent on the continued commitment by State and District level partners. Challenges arose in the intervention during a period when government funding was unavailable, and this lack of funding impacted the availability of peer outreach activities, condom availability, and clinic attendance rates. Although the intervention itself does not rely on additional funding, the feasibility and scalability of the DIFFER model depends on the continued support of governmental-level stakeholders of existing targeted interventions. Ashodaya has worked closely with stakeholders throughout the course of the intervention. These stakeholders have found the DIFFER approach to be highly reliable and felt this should be scaled up, indicating their likely continued support.

### Limitations

This study used a pre-post test design and lacked a control group. However, Ashodaya’s long history of working with women in this setting and comparisons to previous program data provides important insights to interpreting the impacts of the DIFFER intervention on SRH services access. A respondent-driven sampling strategy (RDS) was applied to allow comparisons across surveys and improve the inclusion of sex workers that might be missed with a time-location cluster sampling approach, such as women who do not recruit clients in publicly visible spaces. RDS is similar to snowball sampling, but corrects for this bias through statistical adjustments that attempt to account for social network size and similarity among persons within social networks [39–40]. In our findings, we present the results adjusted with weights appropriate for RDS sampling. There remains a theoretical possibility that selection bias might have cropped up as a result of a variable reporting of network size by the study participants between the two surveys, thus affecting the real changes in sex work populations. Specifically, in Mysore, many sex workers reported very high network sizes. This is possibly to a large extent due to sex work collectives such as Ashodaya Samithi in Mysore. Nonetheless, we believe that despite a possible high level of interconnectedness, this scenario would not have a substantial impact on our findings, since we see no reason why the level of interconnectedness would change between surveys. Hence, it is unlikely that level of interconnectedness, or any change in that, might be the reason for our results.

### Conclusion

Mysore is characterized by an already well-mobilized sex work community, including a sex work organization, Ashodaya Samithi, with a membership of over 8000 sex workers. Targeted HIV and STI interventions among sex workers, including a sex work-led clinic, have achieved high coverage. DIFFER therefore operated as a ‘diagonal’ targeted intervention, by adding SRH services to a well-established sex worker-led intervention. Findings from DIFFER support incorporating SRH into existing HIV/STI service delivery models. Further work should focus on continuing to improve access to cervical cancer screening services and treatment, as well as advocacy at the local and national levels to support the scale up of SRH and STI/HIV services in an integrated way across the state and country.
